# Review of Explosive Contamination and Bioremediation: Insights from Microbial and Bio-Omic Approaches

**DOI:** 10.3390/toxics12040249

**Published:** 2024-03-29

**Authors:** Daniel Corredor, Jessica Duchicela, Francisco J. Flores, Maribel Maya, Edgar Guerron

**Affiliations:** 1 Departamento de Ciencias de la Vida y la Agricultura, Universidad de las Fuerzas Armadas, ESPE, Sangolqui 171103, Ecuador; fjflores2@espe.edu.ec; 2 Centro de Investigación de Alimentos, CIAL, Facultad de Ciencias de la Ingeniería e Industrias, Universidad UTE, Quito 170147, Ecuador; 3 Departamento de Ciencias Económicas, Administrativas y de Comercio, Universidad de las Fuerzas Armadas, ESPE, Sangolqui 171103, Ecuador; ammaya@espe.edu.ec; 4 Departamento de Ciencias Exactas, Universidad de las Fuerzas Armadas, ESPE, Sangolqui 171103, Ecuador; erguerron@espe.edu.ec

**Keywords:** biodegradation, TNT, soils microbiology, explosives pollution, toxicity, bio-omics

## Abstract

Soil pollution by TNT(2,4,6-trinitrotoluene), RDX(hexahydro-1,3,5-trinitro-1,3,5-triazacyclohexane), and HMX(octahydro-1,3,5,7-tetranitro-1,3,5,7-tetrazocine), resulting from the use of explosives, poses significant challenges, leading to adverse effects such as toxicity and alteration of microbial communities. Consequently, there is a growing need for effective bioremediation strategies to mitigate this damage. This review focuses on Microbial and Bio-omics perspectives within the realm of soil pollution caused by explosive compounds. A comprehensive analysis was conducted, reviewing 79 articles meeting bibliometric criteria from the Web of Science and Scopus databases from 2013 to 2023. Additionally, relevant patents were scrutinized to establish a comprehensive research database. The synthesis of these findings serves as a critical resource, enhancing our understanding of challenges such as toxicity, soil alterations, and microbial stress, as well as exploring bio-omics techniques like metagenomics, transcriptomics, and proteomics in the context of environmental remediation. The review underscores the importance of exploring various remediation approaches, including mycorrhiza remediation, phytoremediation, bioaugmentation, and biostimulation. Moreover, an examination of patented technologies reveals refined and efficient processes that integrate microorganisms and environmental engineering. Notably, China and the United States are pioneers in this field, based on previous successful bioremediation endeavors. This review underscores research’s vital role in soil pollution via innovative, sustainable bioremediation for explosives.

## 1. Introduction

Military activities exert substantial influence on soil properties, primarily through physical and chemical disturbances arising from the use of explosive materials during military training and warfare, therefore, pose risks to both human health and ecosystem stability. A less acknowledged consequence is the disruption of soil structure, which can severely compromise properties, such as hydraulic conductivity, leading to environmental challenges like heightened erosion [1] and chemical imbalances [2].

Upon detonation of demolition artillery rounds, warfare mortars, and hand grenades, a relatively small percentage (10^−3^ to 10^−6^%) of the initial explosive remains in the soil surface as chunks, slivers, and soil-size particles (<2 mm) [2]. Furthermore, ammunition components adjacent to low-order detonation sites can reach abnormal and harmful concentrations (hundreds to thousands of mg/kg) in soil [3]. The deflagration process often leaves behind residual pollutants, as the complete reaction of explosives requires a high energy input. If that energy is not reached, incomplete or partial reaction occur [1], it can lead to the deposition of explosives or their derivatives, which are toxic, within soils [4], potentially causing the migration of harmful compounds into groundwater through processes of sorption and desorption, subsequently endangering both human life and the environment [5].

In military explosives and civilian purpose-regulated explosives (as demolition), two primary categories exist, high explosives and low explosives. High explosives are characterized by compounds with low melting points and high thermal stability, and they are commonly referred to as “detonating explosives” [6]. Among these high explosives are notable constituents such as 2,4,6-trinitrotoluene (TNT), hexahydro-1,3,5-trinitro-1,3,5-triazacyclohexane (RDX), and octahydro-1,3,5,7-tetranitro-1,3,5,7-tetrazocine (HMX), and nitrate esters, all of which exhibit high explosion velocities above the speed of sound, typically falling within the range of 5500 to 9000 m s^−1^. On the other hand, low explosives undergo a slower reaction, with a burn rate between 171 and 631 m s^−1^, known as deflagration [5,6]. If the ignition process, which encompasses deflagration and the immediate subsequent detonation, is not strong enough, nitrogen-based explosives are left as residuals. This produces major alterations, soil toxification, phytotoxicity, and microbial disbalance, which alters nutrient intake in soils and roots [7].

Residual TNT, HMX, and RDX persist in soils after firing range exercises and controlled explosive detonations in civilian activities. Using analytical chemistry methodologies, a range of compounds, including TNT, 2,4-DNT, 2-ADNT, 4-NT, TNB, RDX, and HMX, could be detected at concentrations of 0.015–0.190 ppm within a radius of 30 m from the explosion center [2,8]. The persistence of nitrogen compounds explosives can disrupt microbial metabolism and communities through processes of sorption and desorption in soils and water [9]. TNT, HMX, and RDX are often associated with problematic contamination issues and often degrade slowly in environmental systems. Toxicity and mutagenicity of these compounds are widely known, especially in soils. Long-term exposure to TNT, RDX, or HMX has been observed to cause a significant loss in microbial activity and populations. Microorganism adaptation is critical for their survival in soils that are contaminated with nitrogen residual, especially RDX, TNT, or HMX. Soil perturbations caused by land movement (mining, firing, and others), increased porosity, and chemical alterations further amplify the challenges to maintain microbial communities after explosions [3] as such contamination not only affects immediate surroundings but can also impact broader areas due to soil displacement and particle dispersion [9,10].

Studies aimed at improving the bioremediation of soils and water contaminated by explosives have identified specific microbial agents involved in the degradation of these compounds [5,10]. These studies have introduced new microcosm systems capable of reducing toxicity and reshaping microbial dynamics, inducing shifts in the gene pool and modifications related to amino and nitro reduction processes [11]. Research has identified common ancestral genes between microbial genera of fungi, bacteria, and archaea in diverse microbial populations capable of degradation, even in the presence of inhibitors [10,11]. The alteration of microbial community dynamics due to ecotoxicity significantly influences microbiota proliferation in soil and the effectiveness of remediation responses [12].

The primary goal of this review is to describe the effects of TNT, RDX, and HMX in microbial communities through a bio-omics perspective, placing special emphasis on the role of mycorrhizae. Additionally, we will examine technological advancements that have led to patented innovations in this field and offer insights into the future prospects of such research endeavors.

## 2. Materials and Methods

### 2.1. Methods Data Extraction and Collection

The paper adopts a systematic review approach including all pertinent prior research within the context of explosive degradation and soil microbiology, aiming to ensure comprehensive coverage of relevant topics, which includes the examination of bacterial consortia, fungi, mycorrhiza, and plants. The foundation of this review rests on principles of repeatability and transparency. Specifically, the selection of articles was confined to peer-reviewed publications and the most recent editions of relevant books. To ensure the accuracy and reliability of the information presented, topics of interest were rigorously assessed for the presence of substantiating evidence, effectively addressing the issues under consideration. The process of acquiring suitable information involves defined steps, which include both inclusion and exclusion criteria.

The first phase of this research involved a bibliometric analysis of databases to identify and focus on key topics related to explosive degradation and soil microbiology. The selection criteria for this phase were designed to capture research that aligns with the primary research goals. The analysis encompassed two prominent databases: Web of Science and Scopus database on August 2023. The time scale of our data is from 1 January 2013 to 20 October 2023. Additionally, English is required to be the publication language. These databases were chosen due to their comprehensive coverage of scholarly literature. The data considered for analysis included working papers, conference papers, book chapters, and various article types while excluding encyclopedia entries and other non-relevant content.

A systematic search strategy was employed using the following keywords: “explosives degradation”, “TNT degradation”, “RDX degradation”, and “HMX degradation”, in combination with terms such as “Microbial”, “recovery”, “bioremediation”, “phytoremediation”, and “fungi”. The search was refined to focus on abstracts, titles, and keywords, optimizing the search for relevance. A total of 162 articles of interest were identified through the combined search efforts in Scopus and Web of Science.

In the second phase, articles were systematically filtered to ensure alignment with the research goals. For data analysis, after downloading the .bib archive of the database, an online/R-package analysis platform of bibliometrics (http://www.bibliometrix.org, accessed on 16 August 2023) was used to quantify the volume of literature in different years, countries, institutions, and journals. This was completed by using the commands, library(bibliometrix) and biblioshiny in R, where the data was loaded. The biblioshyny software was used to analyze cooperation relationships between countries or institutions. This software made the descriptive analysis of a bibliographic data frame, Network creation for bibliographic coupling, co-citation, collaboration, and co-occurrence analyses. Additionally, the biblioshiny software was used to visualize the data network and Mapping. Excel, Citespace (version 5.8 R3), and VOSviewer were used for reference co-citation, author analysis, keyword cluster, and keyword burst analyses. The results from the Bibliometrix analysis, including network co-occurrence and trend analysis, were used to guide the selection process. Additionally, related articles were cross-referenced using Google Scholar to gather further information and insights. Articles that did not directly pertain to the research topics were excluded. This phase led to the exclusion of six articles that did not align with the goals of the review. The results of the bibliometrix analysis indicate that the relevant information on explosive degradation and soil microbiology is concentrated in research from various countries. Moreover, it highlights that the main topics within this field consistently emerge in both network analysis and trend topics.

All titles were assessed for relevance to the systematic review. Articles that did not include terms related to microbial bioremediation, or the specified query search in their titles were rejected. In this phase, 10 articles were excluded, while similar or cited articles were considered for inclusion. Consequently, a total of 104 documents combining articles, reviews, and book chapters, were retained.

After examination of the abstracts and a brief reading of the full texts, articles, reviews, and book chapters that lacked pertinent information were eliminated. As a result of this rigorous assessment, the total number of relevant articles that met the inclusion criteria was reduced to 79. 

### 2.2. Research Methods for Patents

The research focused on identifying patents related to the bioremediation, degradation, or attenuation of explosives such as RDX, TNT, and HMX. The selection process followed specific criteria to ensure the relevance and appropriateness of the patents. The initial step involved searching the Google Patents database using keywords related to explosives and bioremediation. The keywords used included “explosives”, “TNT”, “RDX”, “HMX”, “degradation”, “bioremediation” and “recovery”, in a time frame from 2003 to 2023. Each search yielded more than 1000 patents, the database showed the tendency along time and publication and filled the general Supplementary Materials.

To narrow down the selection, patents that did not meet microbiological criteria or involved non-biological methods were excluded. Additional keywords like “microbiological”, “bacteria”, “plants”, and “fungi” were used to filter out irrelevant patents. The exclusion parameters were applied to reduce the number of patents to be analyzed. The remaining patents were further screened based on their relevance and whether they included genomics analysis or certified microbiological studies. This step ensured that the selected patents aligned with the research objectives. 

Patents were categorized based on patent classifications, including B09C1 (reclamation of contaminated soil microbiologically), C02F3 (related to underground water and persistent pollutants), A62D3/02 (processes for making harmful chemical substances harmless), C12N1 (cultures and fungi), and others, as available. Thus, the final number of relevant patents was 16, and two general articles of patented review in bioremediation.

## 3. Results

### 3.1. The Output of Related Literature

**Figure 1:**
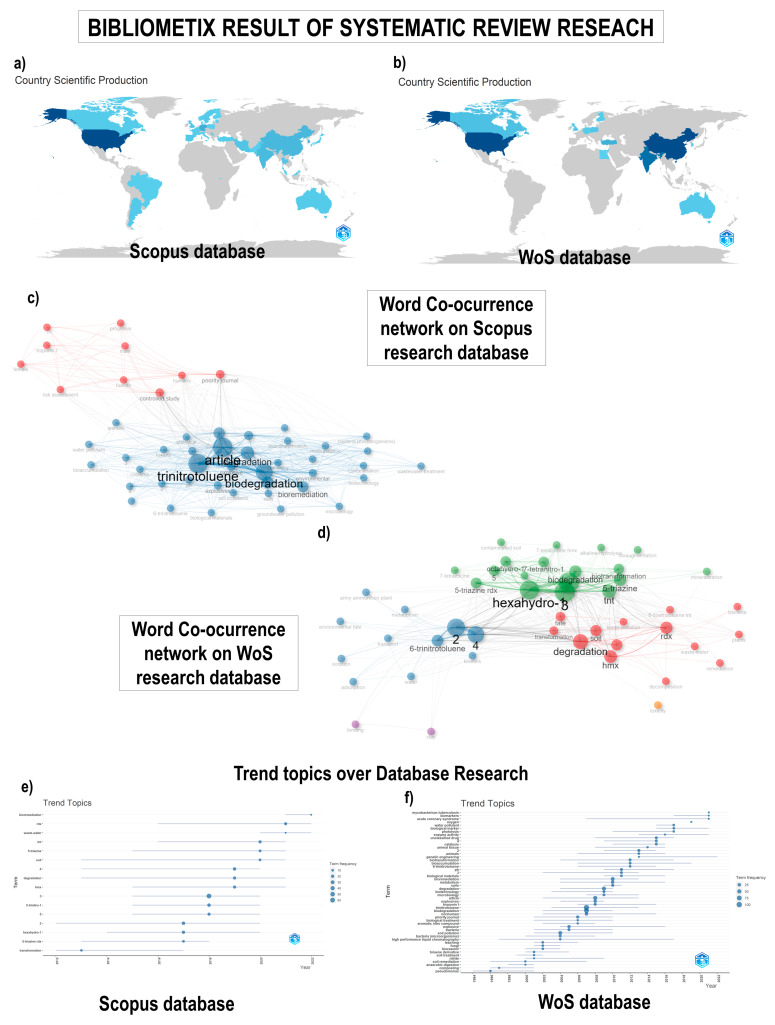
Sistematic review using Bibliometrix. (**a**) Country of Scientific Production of primariy literature published in Scopus databases from 2003 to 2023, countries in blue shows higher scientific production (N° of articles higher than 90) and light blue (N° of articles lower than 24) shows average scientific production. (**b**) Country of Scientific Production of primariy literature published in WoS databases from 2003 to 2023. Blue (N° of articles higher than 35) and light blue (N° of articles lower than 12) shows scientific production (**c**) Word co-occurrence network in Scopus databases from 2003 to 2023. It shows the key concepts and relationships among them. The central keyword is “biodegradation”, with consistent presence of keywords like “soils”, “water”, and “microbiology each color represents a cluster of word coincidence difrenciating 2 groups (red related to keywords of risk, human and journal and blue, related to biodegradation, trinitotuluene and bioremediation). (**d**) Word co-occurrence network in Scopus databases from 2003 to 2023, three cluster of word coincidence are separated by color with nucleous off cluster been, “toxicity”, “hexohydro-1”, “trinitotulene” for blue, green and red respectively. (**e**) Trend topics over database research in Scopus the terms “RDX” “HMX” and “TNT” are the mos frecuent and last over time (**f**) Trend topics over database research in WoS the terms “bacteria” “Biodegradation” and “trinitotuluene” are the mos frecuent and last over time. Database quality and query specificity are crucial in shaping our integrated review.

To conduct a comprehensive analysis of the literature on microbial degradation of munition waste, we obtained related articles from Scopus and WoS, the world’s most comprehensive multidisciplinary literature database. Our inclusion criteria resulted in 79 publications (68 original articles and 11 reviews) from 2013 to 2023 (Figure 1). The number of publications per year is presented in Figure 1 as trend topics over database research and country scientific production, indicating a rising trend with slight fluctuations over the past 6 years.

A total of 25 countries have been involved in this field, with the United States ranking first on the list, having published 98 articles, accounting for 92% of the total publications. Ranking second place is China, with 17 publications (Figure 1). 

Keyword analysis is a crucial step in identifying research hotspots and predicting future directions for new researchers. In this study, we evaluated keywords in four aspects, with the aim of providing a comprehensive picture of research trends. We have tallied the frequency of keyword occurrences and identified the top five keywords for analysis (Figure 1) in trend topics over database research. The keywords with the highest occurrence rates were “Trinitotuluene”, “Biodegradation”, “Article” and “Explosives”. Inboth databases, the plot differs in the structure and the co-occurrence network due to the article focus and search engine.

### 3.2. Impacts of Explosives Pollution on Microbial Community Dynamics

Pollution caused by explosive use, both from military and civilian activities, has been documented since World War I. It has been proved to cause long-lasting and detrimental effects on soil. The decomposition of explosive compounds generates hazardous, toxic, and mutagenic by-products, requiring soil and groundwater remediation [9].

In military training grounds or active demolition zones, persistent explosive contamination of soils (including RDX, HMX, and TNT) has been observed, resulting in a suppression of microbial activity. This suppression may be attributed to the elevated concentrations of explosives and their combined effects, along with other contaminants [2,5]. Contaminated soils, exhibit sensitivity to nitroaromatic compounds, leading to shifts in the gene pool and metabolism in microorganisms [13]. Alterations due to these shifts in the microbiome tend to favor the proliferation of adaptable organisms while reducing toxicity for less adaptable microorganisms through extracellular reduction processes [13,14]. The processes involved in adaptation include enzyme active site reconfiguration, the introduction of modified proteins, and even new metabolic pathways, which in standard conditions are only for nitrogen uptake and transformation. The additional steps required for nitramine degradation and mineralization represent a critical bottleneck in the process, demanding energy consumption and microbial adaptation [15,16]. 

Factors such as pollutant chemical reactivity, phase distribution, in situ inhibitors, competition for oxygen, and leaching directly impact the indigenous microflora community. Moreover, the stability of the indigenous microbiome depends on interactions involving metabolites and nutrient sources [11]. The presence of explosive toxic compounds and by-products in soil obliges a concerted effort to stimulate the proliferation of amino and nitro reducers. Many microorganisms struggle to survive in soils contaminated with nitroaromatic explosives due to their toxicity, which is often linked to mutagenic effects, alterations in membranes, or disruptions in normal metabolic pathways [14,17]. This is a crucial measure to mitigate the toxicity of explosives and restore microbial balance in the soil, which is a vital factor for soil health, crop cultivation, and sustainable natural resource utilization [15,17].

Changes in microbiome composition in tilled and untilled TNT-contaminated soils within military range areas have been linked to biodiversity loss and alterations in soil properties. 16S rDNA sequencing data reveals a shift in the percentage of genera, with *Proteobacteria* comprising 47.2 ± 13.2% and *Acidobacteria* at 25.9 ± 11.8% in the tilled TNT soils, whereas, in nearby contaminated soils, more than 90% of the sequences belong to *Proteobacteria* [13,18]. Research in co-polluted explosive soils indicates a higher abundance of non-common microbiota (such as *Achromobacter*, *Phaeospirillum*, *Thiobacillus*, *Microbacterium*, etc.), representing 31.5 ± 3.3% of the detected sequences, compared to 4.3 ± 2.1% in untilled or non-detonated adjacent areas [19]. This shift may be attributed to the necessity to adapt and claim new ecological roles, but generates a clear decrease in soil quality and the loss of niche diversity, impacting land use. 

From a microbial perspective, explosive pollution, characterized by high-energy nitrogen compounds, exerts a significant influence on microbial communities, this phenomenon leads to an exponential increase in environmental DNA related to certain microorganisms like *Pseudomonas*, *Arthrobacter*, *Archaea*, and *Geobacter*. Initially representing less than 1% of the sequence pool, these microorganisms can account for at least 15% of all annotated sequences [13] in controlled and monitored fire shooting and military practice areas. Soils require diversity, and the dominance of specific genera can disrupt this balance, ultimately leading to poor soil quality in the long term. 

The loss of microbial diversity affects nutrient cycling and land use, while disturbances in geochemical properties result in significant soil degradation. Warfare ammunition and detonations induce changes that harm critical aspects of soil quality and overall wellness. Once pollution occurs, the goal is to achieve complete or near-complete recovery of the soil’s previous characteristics. Biological-mediated remediation, particularly microbial bioremediation, is a promising approach when high-energy nitrogen compounds or xenobiotic pollutants are the primary contaminants. 

### 3.3. Microbial Explosive Degradation and Bioremediation: Advantages and Challenges

In response to environmental pollutants, ecosystems naturally adapt to mitigate their impacts. The concept of bioremediation represents a controlled process aimed at detoxifying and restoring the environment to its previous state [20]. In recent years, various pathways for achieving bioremediation have been described, with a focus on maximizing efficiency. 

**Figure 2:**
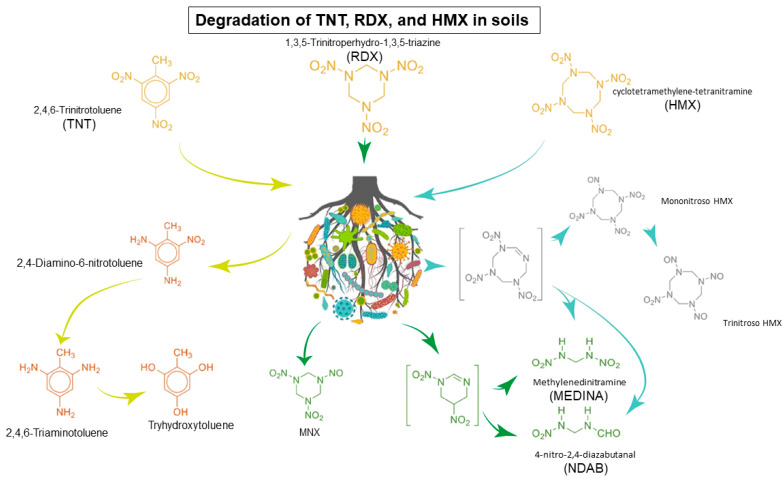
Summary scheme of metabolism of natural environment contaminated with TNT (bright green), RDX (dark green), and HMX (pale green) in soils. Derivatives, and intermediates (in brackets) [27].

TNT, RDX, and HMX are high-energy ring compounds, making them potent explosives and energy sources. Bioremediation and elimination strategies in polluted soils primarily center on nitrogen uptake as the sole source of this element to remove the pollutant, leaving only non-substantial traces of the nitrogen aromatic ring in the case of TNT, RDX, and HMX. These explosives exhibit both anaerobic and aerobic degradation pathways, offering flexibility in bioremediation approaches. For example, TNT produces several nitro-compounds, with 2,4-diamino-6-nitrotoluene (2,4-DANT, see Figure 2) being a common nitro-reduction product. This compound can enter normal metabolic pathways or participate in various metabolism cycles, enabling many microorganisms to achieve detoxification and mineralization of the pollutant [21,22].

The chemistry of RDX is complex, with the main compound aromatic ring containing a cleavable nitro group that increases its stability and energy during deflagration [12]. Therefore, RDX is recalcitrant, and numerous degradation pathways have been identified, primarily involving specific microbial strains. For instance, both aerobic degradation and alkaline oxidation lead to the production of 4-nitro-2,4-diazabutanal (NDAB) [23,24], this compound is toxic, producing a lack of microbial proliferation, metabolic alteration, and environmental stress, as a matter of fact, in RDX biodegradation this compound could be used as carbon and nitrogen source. Nonetheless, due to the necessary energy to start biotransformation, this process is challenging, so in order to reduce unnecessary efforts and avoid adverse effects, such as adaptative response to pollution, responses like the development of more efficient enzymes and expression of new/modified genes taking current metabolic pathways are used to overcome RDX degradation issues. Additionally, the environmental impact of metabolites like MEDINA and NDAB (RDX derivatives, see Figure 2) can be more recalcitrant and have a harder way of treatment than the initial compound, requiring a reductive denitration for complete mineralization. Bioremediation can mitigate stress and toxic effects by fostering beneficial interactions within microbial consortia or modifying metabolites [25]. 

HMX degradation produces highly toxic nitrogen molecules (n-hydroxymethyl-methylene-dinitramine and methylene-dinitroamine, see Figure 2) that dysregulate genes related to amino acid biosynthesis and various metabolic pathways. These pathways play a role in resisting abiotic stress and reducing toxicity [26]. Bioremediation of RDX or HMX often involves an adaptation phase where modified pathways, proteins, and genes work to mitigate the pollutant’s impact.

The use of microbial resources in remediation offers several advantages, particularly in regions affected by detonations or demolitions and warfare activities, where ecosystems are fragile and damaged. Non-microbial techniques can be disruptive, in soils that possibly will be used for another purpose, whereas biological treatment is less intrusive and facilitates detoxification and environmental recovery without causing additional harm [27]. This approach is scalable, cost-effective, and can achieve up to 90% degradation and detoxification [28]. Moreover, the adaptation and development of self-cleaning mechanisms by explosive-degrading microorganisms offer long-lasting effects and resilience against future pollution events (Table 1).

The advantages of bioremediation often outweigh the disadvantages in terms of cost. Chemical or metal oxide-reduction degradation may be cheaper (20–40% overall) [29], but biological degradation remains a necessary step to fully mineralize by-products. Combining these approaches ensures remediation without prolonged adaptation or significant stress on the soil microbiota [20]. Bioremediation techniques are adaptable to soil requirements and can be tailored to address specific microorganism-related treatments or other exigencies. As shown in Table 1 prior to implementing bioremediation, studies must be conducted to comprehensively address degradation and environmental recovery [5]. Recent studies related to bioremediation, molecular tools, and bio-omics sciences have provided crucial evidence to support or refute specific processes. The subsequent section will explore and analyze RDX, TNT, and HMX pollution from the perspective of molecular tools and bio-omics science. 

**Table 1:** Summary of studies on microbial explosive degradation and bioremediation of TNT, RDX, and HMX in soils.

Title	Munition Waste	Organism	Species ID	Method	Removal Efficiency	Timeline
Ruminal bioremediation of the high energy melting explosive (HMX) by sheep microorganism [30].	HMX	Bacterial strains from consortia of ruminal fluid	*Anaerovibrio lipolyticus*, *Butyrivibrio fibriosolvens*, etc.	strains in culture with liquid media with 17 µM of HMX with low carbon and nitrogen basal media, after an analysis of HPLC in days 0, 1, 4.5 of incubation.	90% of all HMX derivatives	In 120 h, the 5 first hours show quickly metabolic change in HMX, later only minor Metabolist found by HPLC by-products.
Anaerobic biodegradation of RDX and HMX with different co-substrates [31].	HMX, RDX	Bacterial granular sludge	*-*	In vitro anaerobic treatment of 33 mg L^−1^ for each HMX and RDX in water with co-substrates such as such as ammonium chloride, dextrose, sodium acetic, sodium nitrate and sulfate, degradation measured through HPLC within 10 days.	99.1% and 98.5% in carbon co-substrates after 7 days	In 10 days of anaerobic degradation, RDX concentration decrease quickly than HMX in al co-substrates, in short period of time the study shows kinetics of degradation with monitoring every day.
Evaluation of biostimulation and bioaugmentation to stimulate RDX degradation in an aerobic groundwater aquifer [32].	RDX	Bacteria	*Gordonia* sp. strain KTR9	In situ remediation by biostimulation (fructose injected) and bioagugmentation (cell injection) in groundwater, with Bioaugmentation treatment costs which is estimated at ~$250 in contrast of 85$ for only biostimulation.	80% and coefficient rate of 1.2 day^−1^ For bioaugmentation and 0.7 day^−1^ for high carbon biostimulation	In third spatially time experiments within 150 days, monitoring of RDX levels and bacterial UFC were a main focus, increase and decrease of bacterial strains after and before remediation techniques were measured giving a good timelapse of this experiment and fidelity of data.
Enhancing the Potential for in situ Bioremediation of RDX Contaminated Soil from a Former Military Demolition Range [33].	RDX	Indigenous bacteria for soil samples	*-*	A column of soil extracted from a former military demolition site and the use of use of waste glycerol for in situ treatment of soils contaminated with energetic-materials, measure with HPLC in the important sites of soil column with 600 mg of RDX.	95% of removal in 2 months and 99% with the use of glycerol	The timelapse of 40 days offers whole monitoring of RDX, and the data is used to scale-up.
Passive in situ biobarrier for treatment of comingled nitramine explosives and perchlorate in groundwater on an active range [34].	RDX, HMX and perchlorate	Biobarrier microorganims	*-*	A bio-barrier designed for underground water leaked of explosives, injected oil in barrier a well for stimulates the degradation and concentrations of toxic pollutants were for the groundwater stream.	removal averaged 83 ± 17% for the in-barrier wells and 75 ± 21% for the centerline *	In 1000 days of monitoring this timeline of toxics pollutant were collected and give a clear perspective of treatment evolution barrier development and degradation rates.
Spatially-distinct redox conditions and degradation rates following fieldscale bioaugmentation for RDX-contaminated groundwater remediation [35].	RDX, HMX and NDBA	Bacterial strains	*Gordonia* sp. KTR9 and *Pseudomonas fluorescens* strain I-C	A push up test in well bioaugmentaion for groundwater and soil in expression of *XplA* and *XenB* genes. approach to real in situ treatment and long-lasting effect in soil and groundwater.	RDX cleanup level of 0.8 μg L^−1^ in less than 10 years	The timeline explains years of well degradation of HMX and RDX and by-products like NDAB, microbial status and biogeochemical conditions are monitored constantly in during the study.
Phytoremediation of multiple persistent pollutants co-contaminated soil by HhSSB transformed plant [36].	TNT	Plant	*Halorhodospira halophila*	The heterologous expressing *Halorhodospira halophila* single-stranded DNA binding protein gene (HhSSB) improves tolerance to 2,4,6-trinitrotoluene (TNT), 2,4,6-trichlorophenol (2,4,6-TCP), and thiocyanate (SCN−) in *A. thaliana* and tall fescue (*Festuca arundinacea*).	TNT and by-products were reduced to 1.63 mg/kg (up to 92% of uptake) **	In timeline the 30 days are a major advance in degradation and change in plant DNA expression and toxicity tolerance through growth and develop in plants.
Enhanced phytoremediation of TNT and cobalt co-contaminated soil by AfSSB transformed plant [37].	TNT	Plant	*Acidithiobacillus ferrooxidans*, *Arabidopsis thaliana*	The plant’s root length was measured after TNT or CoCl_2_ exposure to determine their tolerance to TNT and Cobalt.	TNT reduced to 1.23 mg/kg (up to 95% of efficiency uptake) **	15 days are enough time for observe degradation and change in plant DNA expression.
Bioremediation of Explosive TNT by *Trichoderma viride* [38].	TNT	Fungi	*Trichoderma viride*	Growth and toxicity resistant in vitro of soil fungi able to overcome the nitrogen uptake from a toxic pollutant as TNT, degradation levels measured by chromatography with 100 ppm of pollutant.	90% or less in terms of low concentration in plate	In 11 days, the monitoring of fungi plate growth shows, real degradation and byproducts meanwhile the organism persist and adapt.
Optimization of process parameters for degradation of HMX with *Bacillus toyonensis* using response surface methodology [39].	HMX	Bacteria	*Bacillus toyonensis*	In vitro response of isolated strain in Response surface methodology for toxic pollutant with 2–6 mg L^−1^.	87.7% degradation was achieved at 2 mg L^−1^ initial HMX	In timeline of 15 days the result described real degradation and inhibition of growth. Monitoring indicates inoculum direct viability dependent of concentration.

* The efficiency of the biobarrier varies in dependence of flow and other factors, efficiencies rates must be compared with criteria compared in the field well or soil studies, also in biobarriers or other similar treatments the description of all microbiota is not described by authors in general. ** In pants removal efficiencies and uptake are mixed due to the nature of the bioremediation process and certain levels and concentrations depend on the result focus.

## 4. Bio-Omoics Approach and Molecular Tools in Bioremediation and Pollution

Bioremediation processes require comprehensive analyses to gather data on the specific case. In this context, the use of metagenomics, transcriptomics, proteomics, metabolomics, lipidomics, and epigenomics—collectively referred to as bio-omics—provides a molecular and genetic approach. This approach allows for gainining insights into pathways, identifying the most efficient organisms, predicting future profiles, and achieving remediation goals. These studies serve as essential tools for understanding the extent of pollution damage and establishing the focal points for remediation. In this section, bio-omics will provide a clear picture of explosive pollution and its degradation.

### 4.1. Metagenomics for Functional Insights

Metagenomics, the study of the structure and function of whole genomes in a sample of isolated genetic material, is a crucial tool for understanding the distribution of organisms in soils [40]. It plays a vital role in bioremediation, pollution assessment, long-term monitoring, and other soil-related technologies. Metagenomic profiling in soils contaminated with TNT, RDX, or HMX pollution often reveals a higher abundance of bacterial genera, such as *Pseudomonas*, *Enterobacter*, *Rhodococcus*, and *Mycobacterium*. In various soils, this dominance of biological and metabolic activity is accompanied by unidentified taxa [40], and different metabolic profiles are linked to nutrient richness, potential land uses, and future environmental cycle recovery.

Metagenomics analysis provides valuable insights into changes and the effectiveness of pollutant removal. For example, in cases of mixed explosive pollution, bacteria from the genus *Rhodococcus* significantly decrease in tilled ammunition zones, while non-dominant genera like *Pseudomonas*, *Stenotrophomonas*, *Geobacter*, and *Agrobacterium* become the most abundant in post-treatment soils. This shift corresponds to an increase in certain phyla or classes within *Proteobacteria* [11,12]. The diversity and abundance of functional genes and bacteria associated with RDX degradation demonstrate significant differences at contaminated sites [41].

Functional gene and microbiome analyses using metagenomics can help monitor for early intervention in pollutant events. Metagenomic studies have revealed that in the presence of TNT, RDX, or HMX, rich and diverse microbial profiles tend to be replaced by less diverse profiles that exhibit gene pools that are favorable for the degradation of these toxic compounds [42,43]. These less diverse profiles are dominated by *Proteobacteria*, *Actinobacteria*, and *Gammaproteobacteria* [44], which may serve as indicators of pollution and toxicity in the soil. Co-occurrence microbial networks in metagenomics illustrate relationships between newly established species after soil treatment and genes related to nitro reduction and other metabolic adaptations for xenobiotic degradation [41]. Interactions, both negative (competition, interference, predation, and others) and positive (cooperation, mutualisms, cross-feeding, and commensalism) between bacterial taxa become intense in the presence of mixed explosive pollutants and their metabolites [44]. 

The power of genomics tools in providing valuable evidence and insights is exemplified in a long-term study conducted in Puerto Rico by Lizbeth Davila-Santiago and collaborators in 2022, historically, human-impacted soils by military activities in Puerto Rico lagoon were dominated by *Bacteroidetes* (68%) and *Proteobacteria* (29%) [45]. Metagenomics analysis of these soils revealed the presence of human gut-associated Bacteroidetes genera, including *Bacteroides, Prevotella*, and *Gammaproteobacteria.* This in-flied study has the traceability of two specific samplings in a period from 2005 to 2014, with greater microbial diversity in an area not affected by explosives used as a control, unlike the impacted site with a clear change in genomics even years after the initial contamination event. Genomics toll used demonstrated the ability of certain genera to resist the stress caused by nitro compounds, indicating genes capable of biodegrading the residuals of military ammunition discharge [46], these genes decreased 91% of their expression and relative abundance in 10 years after the event [45].

Metagenomics serves as a foundational tool for studying soils, it represents just the initial step in a broader investigation. To gain a comprehensive understanding of soil dynamics in response to pollution, other molecular characteristics such as transcriptomics and additional complementary molecular tools should be explored [46]. The use of multiple molecular approaches allows researchers to cover the entire panorama of soil responses and adaptations to environmental stressors, providing a more holistic view of the case status. These tools collectively offer valuable insights into the complex interplay between microorganisms and their environment, shedding light on their roles in bioremediation and ecosystem recovery.

### 4.2. Transcriptomics for Gene Expression Analysis

Bio-omics relies on transcriptomics to assess metabolic pathways and gene expression, which are critical for understanding adaptation, changes, and new dynamics in microbiomes in response to explosive pollution. Transcriptomics complements metagenomic profiling by offering a deeper understanding of the microbiota’s capacity for xenobiotic degradation, mineralization, and the reduction in toxicity caused by explosive pollution [47].

For RDX bioremediation, few genera and strains can oxidize or reduce specific intermediates like MEDINA or NDAB. *Methylobacterium* sp. JS178, through nonspecific nitro-reductases and other enzymes, plays a crucial role in this process [23,48]. Studies suggest the involvement of proteins like *XplA* and *XenB* in various organisms, including *Gordonia* KTR9, *Rhodococcus*, and *Pseudomonas*, in both aerobic and anaerobic pathways related to MEDINA degradation in-situ with efficiencies of 5–11% with an initial 20 mg L^−1^ of RDX. These proteins were detected in locations where 16S metagenomics sequences potentially indicate the coexistence of a diverse community of RDX-degrading bacteria, no matter the geographic zone or relevant differences between the adaptation process [11,23,49].

Transcriptomics is particularly valuable in assessing the response of microorganisms to highly toxic pollutants like RDX and HMX. It sheds light on the dysregulation of metabolic pathways and gene expression, revealing how microorganisms adapt to survive and proliferate under stressful conditions and face the challenges through time caused by these pollutants [47]. In the presence of HMX pollution, microorganisms may experience disruptions in important metabolic pathways like purine, amino sugar, and nucleotide sugar metabolism [50].

Studies focusing on degradation processes, utilizing both metagenomics and transcriptomics approaches, highlight positive interactions (cross-feeding and mutualism) between various microbial organisms involved in biotransformation. For example, degradation involves cross-feeding and co-metabolism) between genera *Desulfovibrio*, *Paenibacillus,* and *Tepidibacter*, succeeding in mineralizing HMX (26.8% in 308 days) leading to an increase in metabolites related to nitrogen uptake [42]. Interactions like metabolite exchange, metabolite conversion, signaling, and chemotaxis, between bacteria contribute to the transformation of compounds like dinitrotoluenes and cyclic nitramines through dioxygenase and denitration mechanisms [16]. 

In a case study focused on the biodegradation of TNT by *Citrobacter* sp., both proteomics and transcriptome analysis were conducted. This study revealed the upregulation of 308 genes, resulting in the degradation of more than 76% of the initial concentration in 12 h, with N-ethylmaleimide reductase (*NemA*) being the most highly upregulated gene. *NemA* plays a critical role in transforming TNT into 4-amino-2,6-dinitrotoluene (4-ADNT) and 2-amino-4,6-dinitrotoluene (2-ADNT) [51]. The upregulated genes were associated with various processes, including energy production and conversion, amino acid transport and metabolism, posttranslational modification, protein turnover, and chaperones. Conversely, 234 genes were downregulated, mainly related to carbohydrate metabolism and transport [51].

Transcriptomics and proteomics are closely interconnected, as changes in gene expression directly influence protein levels and functions. The bio-omics approach aims to extract and integrate data from these molecular processes to comprehensively describe and explain the biological mechanisms underlying biodegradation. Once transcriptomics has been thoroughly explored, the next step is typically proteomics analysis to provide a more complete understanding of the biological processes involved.

### 4.3. Proteomics and Metabolomics Applications

Proteomics and metabolomics are essential tools in understanding and studying the bioremediation of explosive pollution. These molecular approaches are specific to either individual strains or the entire microbial community, providing a comprehensive view of the metabolic changes necessary for xenobiotic degradation. 

One of the major challenges in explosive pollution is dealing with both nitroaromatic compounds and heavy metals, which are often present together in ammunition. Heavy metals like copper, zinc, or lead are components of explosives and ammunition [52]. After detonation, these heavy metals remain in the soil along with the nitroaromatic compounds. Consequently, the genes involved in TNT, RDX, and HMX degradation are often associated with genes responsible for heavy metal detoxification and cytochrome genes to overcome toxicity [44].

A proteomics analysis by Feng et al. showed that cytochrome P450, along with aromatic dioxygenases, oxidizing enzymes, methyltransferases, and general transporters might have an essential role in the degradation of the aromatic toxic compound triphenyl phosphate [53]. Furthermore, a unique variation of cytochrome P450, the *XplA*, an unusual protein structural organization comprising a heme domain, by reductase activity achieves denitration and reductive biotransformation of RDX using flavodoxin as a reductive helper [54]. Proteomics also provides insights into the structural changes in proteins and how they adapt to the degradation process to cope with xenobiotic pollutants.

Proteomics analyses can vary, from studying a single organism or strain to conducting differential zone analyses. For example, proteomic research in *Yarrowia lipolytica* revealed the expression of proteins related to membrane-bound oxidoreductases, which play a pivotal role in triggering the transformation of TNT [55]. This information sheds light on the biological degradation of explosives in plants and fungi and highlights the complex changes in proteins during pollutant bioremediation, even so one limitation of proteomics analysis is its focus on individual organisms or strains, and understanding the proteomics of an entire microbial community remains a challenge [55,56].

An interesting aspect of proteomics and metagenomics is the intricate network of proteins and enzymes involved in the degradation of xenobiotic compounds [51,57]. Adaptable microorganisms in soil microbiota are often associated with multiple co-occurrence network modules suggesting that explosives or toxic pollutants require a higher level of protein expression. Towards efficient degradation, proteins change or adapt active sites, reductive potential, mixed functional units, or structural configuration for xenobiotics biotransformation. These changes alter the relative abundance of genes, the relative abundance of microbial species, and the proliferation of one certain genera above others. The perspectives of these changes, related to all microbiota and expressed proteins, could be explored further in metagenomics studies [29].

Molecular tools, including those involved in proteomics and metabolomics, have played a significant role in achieving a deeper understanding of metabolism and bioremediation as is exemplified in Table 2. These tools have been crucial in advancing our knowledge of the impact of explosives on the environment and driving innovation in bioremediation strategies. In the next section, we will explore innovations in the field of explosive bioremediation.

## 5. Innovative Bioremediation Strategies

Numerous innovative bioremediation strategies are emerging to meet the expectations of efficient cleanup of residues from explosives. These strategies often involve novel organisms with simplified engineering approaches and fresh perspectives. The following section describes these innovative approaches in detail.

### 5.1. Phytoremediation and Rhizoremediation 

Phytoremediation is a technique that harnesses the natural abilities of plants to take up, degrade, and metabolize xenobiotic compounds like those found in ammunition, such as TNT, RDX, or HMX. This approach has proven to be a sustainable and reliable method for rehabilitating polluted areas, ensuring their long-term use [61]. Various types of plants, both aquatic and terrestrial, have demonstrated the ability to uptake TNT in hydroponic cultures without suffering from major toxicity issues. This indicates their potential for phytoremediation [62,63].

In a successful case of phytoremediation, wheatgrass was genetically modified with bacterial genes (*XplA* and *XplB*) to confer the ability to degrade RDX in vitro, in 12 days of culture, removal efficiencies of the modified plant were 87% while the wild type only removed 67%. Additionally, the introduction of a bacterial nitro-reductase gene (*nfsI*) improved the ability of the plant to withstand and detoxify TNT [64,65]. A combination of phytoremediation and microbiological approaches has shown promising results, with soil microbial activity and extracellular enzyme activities related to the nitrogen cycle being upregulated for high removal efficiencies in a short time (up to 90% in 7 days). 

Field demonstrations have used genetically modified switchgrass expressing *XplA/XplB* genes to enhance RDX degradation in military fire ranges. Modified plants were able to uptake RDX, reaching 0.08 mg g^−1^ in their tissues. Furthermore, in a 3-year field study, plants survived and proliferated, achieving removal efficiencies of 98% [66,67]. A proteomic analysis of vetiver grassroot proteins showed the downregulation of functional proteins related to cellular mechanisms like transcription, ribosome biogenesis, nucleo-cytoplasmic protein transport, protein glycosylation, and translation. Conversely, the ethylene signaling pathway and plant defense-related proteins were upregulated, potentially enhancing the plant’s tolerance to higher, non-phytotoxic TNT concentrations by aiding in respiration and reducing reactive oxygen species [57,67].

Phytoremediation has a significant impact on microbial diversity and profiling, especially in the rhizosphere, where microbiomes contain bacteria with the potential to provide essential ecosystem functions. Different plants, through root exudates or symbiosis, offer benefits such as rapid proliferation, increased biomass, and resistance to both abiotic and biotic stresses [64,68]. Molecular analyses show that phytoremediation leads to the upregulation of soil microbial activity and extracellular enzyme activities related to the nitrogen cycle, resulting in high removal efficiencies within a short timeframe (84% to 95% after 60 days), involving bacteria, such as *Sphingomonadaceae* and *Actinobacteriota*, occupying the soil niche and degrading TNT and RDX, while the *Proteobacteria* and *Bacteroidota* phyla proliferate and increase their activity [67]. 

Field demonstrations have shown that cost-effective crop irrigation systems can efficiently restore soil, making phytoremediation increasingly popular in military fields due to its cost-effectiveness and replicability. Furthermore, healthy rhizobia communities play a crucial role in this process by aiding in nitrogen uptake and reducing toxicity [68,69]. Mycorrhizae, the symbiotic association between roots and fungi, particularly species belonging to *Basidiomycota* and some *Ascomycota*, contribute to soil wellness and promote the proliferation of overall soil health in pollution recovery efforts [70,71].

Mycorrhizae alter the physiology of the host and benefit the plant as a whole, enhancing their efficiency in tolerating or resisting pathogenic attacks and various environmental conditions, including high pollutant concentrations [71]. This phenomenon may explain soil concentrations of over 400 ppm of TNT or RDX without resulting in high toxicity [72,73]. Fungi, such as *Podospora anserina*, produce two N-acetyltransferase (NAT) enzymes that reduce the toxicity of certain compounds, including explosives and xenobiotic pollutants providing a tolerance mechanism in contaminated soil to overcome recalcitrant and harmful pollutants [74]. Fungal species like *Gymnopilus luteofolius, Phanerochaete velutina*, and *Kuehneromyces mutabilis* can produce large quantities of enzymes to remediate soil contaminated with nitroaromatic compounds [74,75].

Rhizoremediation and mycorrhizal use have become cost-effective, suitable, and rapid techniques for remediating soils that require quick recovery and further use. In situ myco-remediation costs only 10–50 American dollars per ton of soil within 60 days of total treatment [69]. Mycorrhiza in military ammunition test and deflagration zones face various stresses related to nutrients, temperature, precipitation, plant pathogens, and the growth of weed plants, hence, they adapt to the contaminated site and stimulate degradation, nitrogen uptake, and several bioremediation mechanisms [69,76]. Additionally, these techniques contribute to the recovery of soil moisture, texture, and properties, extending their effects on the surface and deep within the soil, this long-lasting impact is mainly attributed to the maintenance of indigenous fungal organisms’ adaptation to stress and its effectiveness.

### 5.2. Bioaugmentation and Biostimulation

Bioaugmentation and biostimulation are two distinct techniques in bioremediation, each with its unique approach and advantages. Bioaugmentation involves increasing microbial populations by introducing specific agents to enhance biodegradation. On the other hand, biostimulation aims to improve degradation using existing resources and without disturbing the indigenous microscopic organisms.

In the context of RDX degradation, bioremediation techniques often focus on specific strains to address critical points, while augmentation or stimulation of multiple consortia and genera can fill the niche presented by the affected environment. For example, Khan and collaborators in 2021, proposed a model in which a biostimulant, coupled with *Kinneretia asachharohila* strain 12853, plays a crucial role in creating a robust microbial formulation and enhancing metabolism to achieve suitable removal efficiency (2.4 more efficient in the use of biostimulant an up to 75% of denitration). Specific strains play roles in protecting, boosting, and providing specialized degradation to ensure survival and proliferation [77].

Analyzing the complete microbial profile during biostimulation of RDX degradation is challenging. Wang and collaborators in 2020, showed that biostimulation significantly enriched *Proteobacteria* over 90% of the profile but decreased the population of *Actinobacteria*, especially near the elimination zones after 5 weeks and decreasing RDX to less than 200 ppm. Additionally, the microbes representing various phyla or genera, such as *Bacteroidetes*, *Nitrospirae*, *Planctomycetes*, *Chlamydiae*, *Chloroflexi*, and *Verrucomicrobia*, were severely reduced during biostimulation, indicating a reduction in microbial richness [59,78].

Bioaugmentation involves the use of isolated cultures as enrichment or boosters to enhance removal efficiency. For example, M. Amin and collaborators in 2017, described the use of a surfactant as a bioaugmentation helper along with *Planomicrobacterium flavor*, *Pseudomonas auroginosa*, *Entrobactor asburiae*, *Azospirillium*, *Rhizobium*, *Methylobacterium* and *Pseudomonas* strains with removals of 99.1% [79]. Bioaugmentation modifies microbial protein expression profiles, extracellular or intracellular enzyme activity, and relative abundance. In soils polluted with explosives, the bioaugmentation agent deals with toxicity by compensating metabolic pathways (as reductive denitration, redox sequential steps) to use RDX, TNT, or HMX as a nitrogen source [79].

In practice, biostimulation has often been found to be more efficient and cost-effective compared to bioaugmentation. In a 2016 study by Kao C et al., it was reported that promoting the growth and activity of *Achromobacter* BC09, *Citrobacter* YC4, and naturally occurring consortia [20] through biostimulation tends to be relevant for TNT degradation in contaminated soil. However, in the study of M. Amin and collaborators in 2017, although the efficiencies are enhanced, real high efficiencies of removal up to 90% were after 200 days, accompanied by an increase in cost in long-term applications [79]. These studies open up possibilities for patenting technologies and developing new engineering approaches for cleaner, easier, and more efficient bioremediation processes.

## 6. Currently Patented Process of RDX, TNT or HMX Bioremediation, Degradation or Attenuation 

In the field of military technology and innovation, research and patented concepts hold substantial value. Within the context of soil and groundwater remediation, the importance of patented technologies is a matter of both environmental and public concern. However, certain specific pollutants, particularly those associated with explosives, necessitate further research and specialized legal considerations. It is increasingly common for military forces from various nations to spearhead research efforts in these areas [80].

Soil contamination represents a global environmental challenge, with nitro-amines associated with ammunition serving as a specific concern in certain regions. Consequently, many countries have come together to seek remediation solutions. The efforts to achieve a cleaner environment are typically led by highly productive countries, which are often major sources of pollution. As a matter of fact, patented processes often emerge as collaborative endeavors involving multiple nations. The patents are typically registered in these countries, and the associated research findings and documented information are primarily attributed to them [80].

The United States of America is globally recognized for its robust military force, marked by noteworthy innovations and preparedness. In the past decade (2010–2020), the United States, in collaboration with Canada and China, has taken a prominent position in the realm of patented soil remediation technologies. Highlighted patents within this context concern a reductive biological/chemical treatment process [81].

Numerous patented processes and research initiatives have been released through programs like the US Strategic Environmental Research and Development Program and the Environmental Security Technology Certification Program. These endeavors aim to support technology-based studies focused on the remediation and environmentally friendly passivation or neutralization of explosives [82]. China’s remediation efforts extend to various soil pollutants, including hazardous air pollutants (HAPS), heavy metals, and halogens, with an emphasis on cost-effective patentable technologies to remediate toxic and residual pollutants [83].

Since 2010, there has been a consistent annual average of 200 patents related to bacterial consortia for pollutant degradation and detoxification, as reported by Google Patents. These patents are attributed to various entities in the United States (including MIT and civilian industries), China, and Korea. Notably, over the past six years (2017–2023), the United States has primarily focused on techniques involving bioaugmentation, biostimulation, or electrochemical systems, whereas China has excelled in inventing equipment, adopting combined approaches, and employing bioaugmentation in consortia [83]. 

It is important to note that patenting rules vary from one country to another and are subject to specific regulations. In the context of this research, the United States imposes restrictions, allowing the patenting of isolated strains only if they are novel, non-obvious, represent an inventive step, and are useful or capable of industrial application [84]. Consequently, novel genetic resources are a major topic within patents, specifically in the United States [85]. In contrast, China follows different rules regarding patenting, particularly concerning genetic isolates, China’s National Intellectual Property Administration (CNIPA) includes international depository institutions for biological material samples recognized by the Budapest Treaty. The Center for General Microorganism Administration Committee, which oversees the China Microbiological Culture Collection (CGMCC), serves as a significant repository for patented strains and their mixtures. In this context, in the United States the patenting of bacterial consortia is not allowed. On the other hand, it is possible in China if the bacteria are associated with CGMCC cultures and meet specific concentration and genetic description criteria (Table 3) [85]. 

China’s dominant position in patenting consortia and various microorganisms can be attributed to its keen interest in genetic resources and the ease of access provided by CGMCC [102,103]. This advantage becomes evident when considering that cheaper and novel chemical methods such as nanoparticles and photodegradation may take the lead in other countries where conventional microorganism patents often face challenges related to genetic resources and bacterial restrictions for patenting [85,103]. Furthermore, the complexity of treating explosives as security concerns in countries like the United States makes the patenting process more challenging, requiring a balance between military, public, and genetic certification.

**Figure 3:**
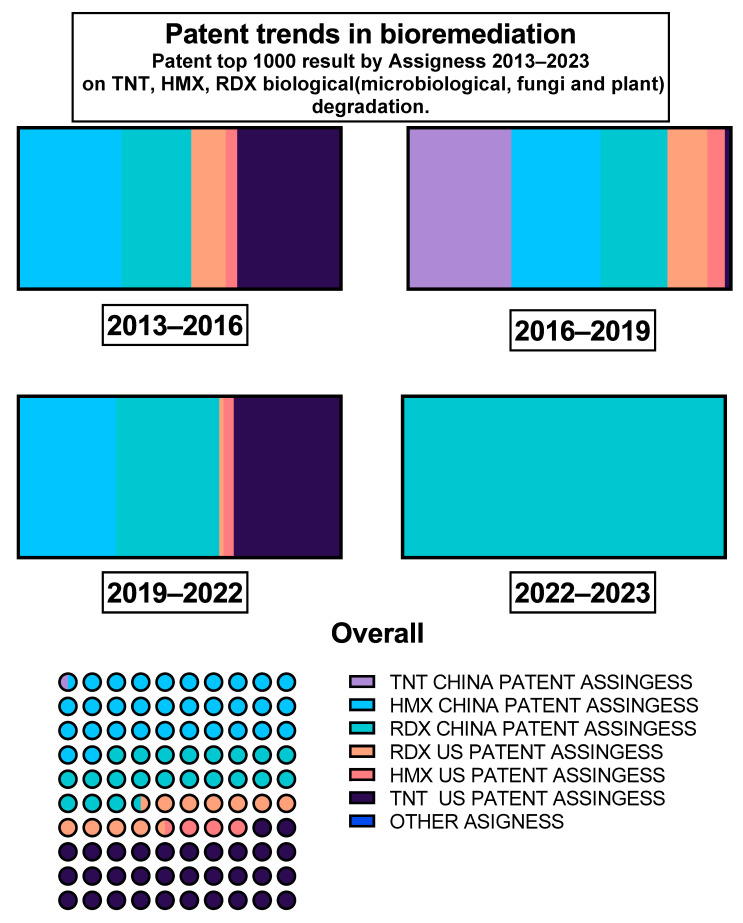
Patent trends in bioremediation related to ammunition damage. It shows China’s increasing focus on this field, whereas the United States has shifted its emphasis from biological to chemical approaches. The United States mainly concentrates on TNT remediation which seems to be widely used for army, demolition, and others industries. Whereas, China explores degradation alternatives on various explosives and mixtures. These trends reflect evolving strategies in addressing ammunition-related environmental issues.

China plays a pivotal role in patent development related to biodegradation, as indicated by Google Patents, where it holds the highest percentage of publications. The Institute of Chemical Defense Chinese Academy Of Military Sciences, in particular, focuses on microorganisms in its patents. China’s industries are also actively engaged in related processes, making it the leading contributor to patent publications from 2016 to 2022. Meanwhile, the United States, led by California University, dominates the previous three years in this domain (Figure 3). 

To provide an overview of the evolving trends in the use of microorganisms and other methods, Table 3 presents a timeline of significant patents related to microorganisms. It highlights the transition over time, emphasizing the increasing prevalence of methods involving consortia of adaptable microorganisms. This approach not only leads to relevant patents but also offers a competitive advantage in the market. Patent innovations primarily revolve around new biological formulations, certified organisms, and applications that address environmental demands, ammunition industry remediation, and the military’s efforts to mitigate damage and restore land use. 

Patents predominantly focus on individual genera or strains rather than consortia. Therefore, bacterial patents related to RDX, TNT, or HMX have a side effect of reducing biodiversity, as the use of one main microorganism the rest of the microbial community must be adjusted to the necessities and surroundings of new conditions of this possibly patented used microorganism. In this context, the relevance is found in the formulation for general bioaugmentation or immobilization of patented organisms and minimizing the spread and change of soil microbial profiles. An essential highlight in this regard is the utilization of previous cultures, resistance genes, or compounds to ensure the bioremediation of xenobiotic compounds (Table 3) [85].

Among the most common bacteria featured in patents, *Pseudomonas* stands out for its adaptability and other traits related to bioaugmentation formulas or toxicity reduction (examples include US2018056.U6(A1), CN102533589(A), CN1043883S2(A), US2010274069(A1), DE102005048904, CN102533589(A), US2018056346(A1), US2018194654(A1), ROI 32554 CAO, AU2016101966(A4), US2010274069, KR 20090030897(A), and KR20080046301). Whereas, bioaugmentation with autochthonous bacteria in soils is rarely employed and is documented in only four well-documented patents covering multiple pollutants (TW2006062S3(A), CN108130291(A), CNIOS85S287(A), MY14S799(A)) [104].

There is an increasing emphasis on fungal applications due to the necessity of soil utilization. Mycorrhiza-based patented processes provide a promising alternative in these cases. According to bibliometric research conducted in 2021, mycorrhizal remediation patents have been granted in 14 countries, with China leading the way (72 patents), followed by the USA (16), Russia, and Korea (6 each) [70]. 

Generally, mycorrhizal patents are associated with biofertilizers or landscape recovery [70]. However, there is no specific data available on the percentage of total patents related to RDX, HMX, or TNT. Nonetheless, it is estimated that 9–11% of patents are related to remediation, and 2–3% pertain to xenobiotic compounds like nitro-amines or ammunition compounds. In this context, mycorrhiza consortium patents are the most prevalent, with China dominating the landscape of mycorrhiza-based patents. 

One noteworthy case involves a strain of *Rhizophagus irregularis* AH01 (deposit number 12157 at CGMCC) [70,104], this strain may have multifunctional applications in mycorrhizal industrial processes and remediation. Patents concentrating on mycorrhizal and fungal use mainly exhibit a prolonged soil treatment, which could prove effective in facilitating nitrogen and phosphorus absorption for xenobiotic compounds. Simultaneously, this innovation may confer certain pathogen and stress resistance due to the fact of the balance in microbiota and metabolic advantages inherent to rhizosphere and symbiosis in certain crops considering metabolomic profiling and microbiota balance [83]. In terms of research and applicants, industry accounted for the largest share of the legal register (45%), followed by universities (24%). Therefore, mycorrhizae represent an industrial subject of interest, and applications in this field may overlap with economic investments [70,81].

## 7. Challenges and Future Directions 

### 7.1. Limited Understanding of Complex Degradation Pathways 

Despite the recognized importance of microorganisms and patented microorganisms in bioremediation, our understanding of their diversity, variety of enzymes, and adaptability remains limited. Understanding of the specific requirements related to biotic and abiotic conditions and their influence on metabolic networks and metagenomic profiles still remains with unclear sections. Bio-omics approaches are focused on elucidating various degradation routes, It is important to note that these descriptions are based on the current soil conditions, including factors like nutrients and moisture [105]. Therefore, a proteomics and metabolomics approach serves as a valuable tool, especially when integrated with extensive databases, to enable more accurate degradation predictions. As described in the Phytoremediation and Proteomics section, it is evident that several biodegradation processes are influenced by intricate extracellular and intracellular interactions such as chemotaxis, signaling, metabolite exchange, cooperation, cross-feeding, etc. Along with other inherent traits specific to microorganisms.

As demonstrated in a 2017 study by M. Kumar and collaborators, even within microbial communities in non-polluted and under non-stressful conditions, there are metabolic pathways and complex co-occurring networks for xenobiotic remediation [106], these networks may play a significant role in the proliferation of communities within contaminated sites and their potential degradative capabilities. However, these metabolic variations can be substantially influenced by both past and current soil conditions [106]. To establish clear remediation techniques and crucial steps to avoid toxicity of pollutants, collaborative effort that involves standardized methods of multidisciplinary science and optimization of current and future patents is mandatory to solve many gaps in our knowledge and efficiency in TNT, HMX, and RDX degradation and remediation. 

### 7.2. Enhancing Biodegradation Rates 

In pursuit of more effective bioremediation strategies, it is crucial to recognize the interplay between initial soil microbial diversity and nutrient requirements, as these factors significantly influence the rate of biodegradation. Many patents emphasize the importance of enhancement methods as a common preparatory step before implementing a bioremediation strategy. This aspect should be explicitly considered when designing bioremediation approaches, including the integration of plants and nutrient supplementation to optimize degradation efficiency and expedite soil recovery.

Patented technologies are in constant competition to achieve cost-effectiveness and greater benefits in the bioremediation process. In spite of a 99% effective process that may remain elusive, enhancing available alternatives represents a practical approach to address desired recovery rates. Recent patents (Table 3) consistently incorporate enrichment agents to expedite mineralization. Among these patents, a combination of initial chemical, photochemical, or nanoparticle degradation followed by microbial processes appears to be the most straightforward and cost-effective strategy [107].

Various external factors can exert significant influence in bioremediation and detoxification, standardized conditions may not apply uniformly to all instances of pollution and degradation. To overcome slow degradation rates and achieve rapid soil recovery, combining methods that complement each other and offer advantages infield could be applicable. For instance, combining rhizoremediation with chemical nitro reduction or phytoremediation with bioaugmentation shows removal efficiencies of up to 95% within low toxic effects and short periods of treatment of 10–40 days [108]. The integration of multiple approaches and the pursuit of non-disruptive patented processes provide valuable alternatives for addressing various challenges that may arise when relying solely on a single, focused method.

### 7.3. Long-Term Monitoring and Risk Assessment 

On a global scale, the establishment of a comprehensive soil database is imperative. This necessitates the collection of standardized soil samples from diverse habitats and as many locations as possible to enrich this database. The ultimate goal is to create a predictive model of soil microbiomes that can achieve high accuracy and forecast critical changes in ecosystem functions [109,110]. Consequently, research efforts will concentrate on enhancing autochthonous microbiota in soils. Strategies focused on expanding land recovery, and even terraforming, to mitigate global climate change are likely to yield higher success rates. However, it is important to note that interaction-based models of microbes and the environment are still relatively imprecise [29].

**Figure 4:**
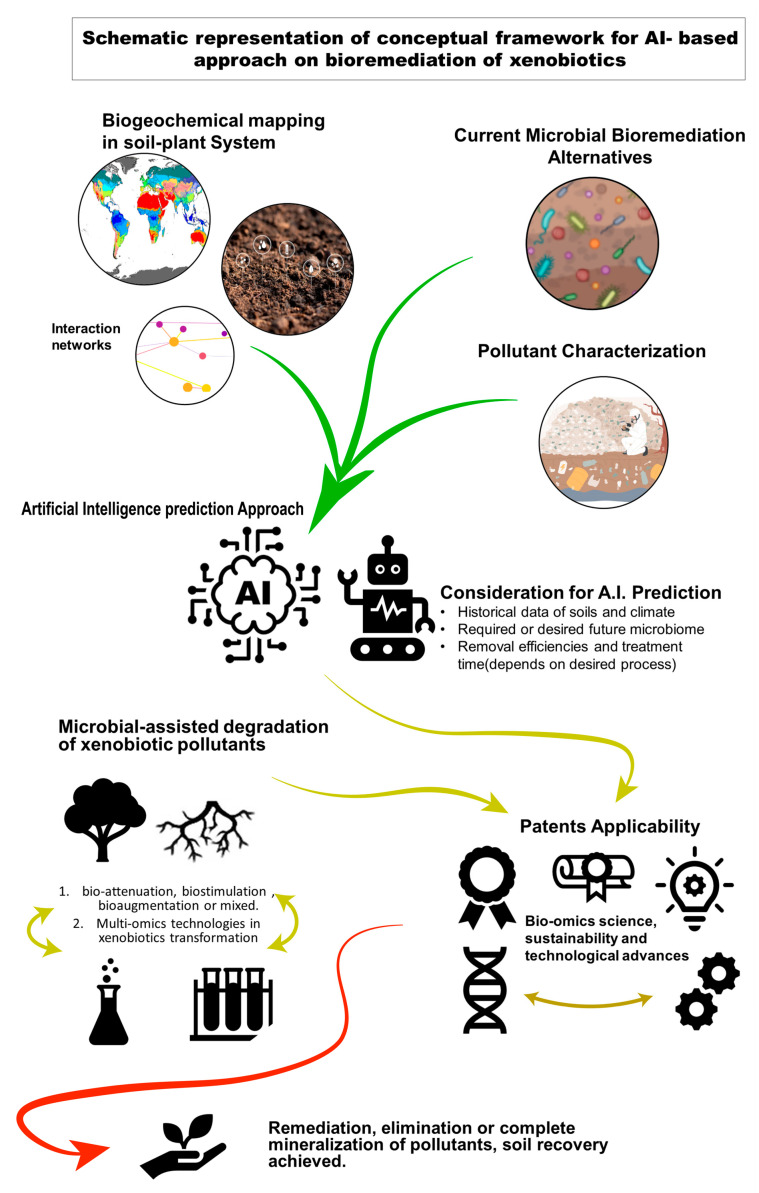
Schematic representation of a conceptual framework for an AI-based approach for addressing environmental damage caused by xenobiotic pollutants. Workflows shows metagenomics and patent processes. The diagram outlines a strategic course of action to achieve optimal results, providing an integrated graphical summary of the topics covered in this article. Ultimately, the goal is to attain sustainable remediation by selecting the most effective patented alternatives andcertified bioremediation processes for explosives bioremediation.

The ability to globally predict future soil biodiversity and ecosystem functions represents a monumental undertaking that requires multifaceted approaches, including biogeochemical, spatial-temporal, and mapping techniques. Nevertheless, we must acknowledge that there exists a gap in our comprehension of the factors and events that influence the transformation of soils [110]. Leveraging techniques such as meta-transcriptomics, metaproteomics, and metabolomics can facilitate predictions of soil microbial communities. These predictions are particularly valuable since soil microbiota are highly sensitive to environmental changes, resulting in alterations to soil profiles through biological and chemical interactions. To address this, the development of a bioclimatic model, based on artificial neural models and multiple interaction networks, provides the ideal input for artificial intelligence to map, predict, and propose sustainable actions that promote both human development and environmental preservation (Figure 4). 

Establishing a comprehensive soil database and the possibility of prediction underscore the importance of microbial-complemented remediation in ensuring the degradation of xenobiotic compounds. Given that ammunition deflagration, military conflicts, and firing areas invariably leave behind damaged or affected zones, in 2022, Kristina Hook described how the soils were affected in the Ukraine war and the poor soil capacity to recover the damage even if the military forces descoped that zone, coupled activities like phytoremediation, biostimulation and bioaugmentation become imperative [111]. Since 2000, the extent of military-affected zones has been expanding, and with ammunition production showing no signs of decreasing, leveraging the microbiome as a rapid tool for soil restoration is of paramount importance.

### 7.4. Integration of Bioremediation with Other Approaches

Metagenomics stands out as a prominent profiling tool in this context. However, substantial efforts are still required in terms of sampling and research to build a comprehensive database of microbial species, which is currently lacking. Future biogeographic studies coupled with metagenomics have the potential to bridge the gap in our understanding of temporal distribution among microbial communities, which remains largely unknown in many cases [109,112]. Achieving a complete comprehension of soil environmental evolution hinges on comprehensive mapping, profiling, and chronological monitoring. Soils evolve over time and utilizing DNA-based approaches (including relic DNA, metabolomics, and cDNA expression profiles) should be aimed at tracking temporal variations in microorganisms or environmental processes, thus facilitating the development of a thorough database [109].

The integrated model, relying on evidence from various bio-omics disciplines (such as genomics and metabolomics), eliminates the need for extensive prior in situ studies following a pollutant event. Additionally, it simplifies the selection of remediation strategies. In the case of xenobiotic pollutants, particularly those prone to easy water infiltration, the utilization of cost-effective materials and non-risky sampling and monitoring methods becomes paramount [29,113]. Consequently, field studies can shift their efforts towards utilizing an evidence-based microbial database to replicate the previous metagenomic profile and subsequently restore the soil [112]. In regions affected by recent fires or military activities, efforts should aim not only at academic achievement but also to establish, whenever possible, the connections and trade-offs between environmental and economic benefits [114].

## 8. Conclusions

In this article, we have conducted a bibliometric analysis of articles related to microbial degradation of munition waste published from 2013 to 2023 and a patent database from 2003 to 2023. The research brings an integrated point of view of microbial use in polluted soils and highlights new developments and alternatives to improve the degradation of explosives like TNT, RDX, and HMX, which has garnered significant interest from researchers worldwide. Notably, studies have focused not only on elucidating the underlying bio-omics (metagenomics, transcriptomics, proteomics, etc.) of the different processes in the biodegradation of residual explosives but also on the use of mycorrhiza and phytoremediation or non-conventional microorganism to assure the soil recovery. 

Over the past two decades, there has been a steady increase in research on this topic, and this trend shows no signs of slowing down. Studies have identified Microbial degradation as the most critical topic for soil management with explosives linking the necessity of efficient processes with patented technology, in this field as the research shows novel information, patents transform and adapt this to useful and practical technology. New research trends have emerged, such as investigating metagenomic and proteomics, which have become the new hotspots in this field.

We believe that further research aimed at elucidating the underlying metagenomics profiles of complex microbiomes in soils, linking the necessity of environmental wealth and sustainable land use, will advance the integration of patented processes related to microorganisms in military fields. This could introduce new solutions for mitigating environmental disturbances after warfare, the production of ammunition, or the military practices that significantly impact the environment. The research will contribute to the development of novel interventions that can be patented and help affected populations or zones that require treatment at low cost and generate the highest reachable benefit in terms of sustainability. Therefore, we urge continued investment in this field to uncover new insights and develop effective treatments, the reason is that the use of explosives will not decrease, and no matter which purpose, the soil will be affected, military activities have a high impact on the environment and need to focus on mitigating that impact for the future land use.

## Figures and Tables

**Table 2:** Summary of studies on Bio-omocs and molecular tools applied to bioremediation of TNT, RDX, and HMX in soils.

Research Title	Insight in Bio-Omics	Efficiency and Remediation Results	Mechanism to Overcome Toxicity	Timeline Changes
Characteristics of RDX degradation and the mechanism of the RDX exposure response in a *Klebsiella* sp. Strain [48]. *	Metabolomic profile of a *Klebsiella* sp. In RDX exposure showing metabolic upregulated genes and the response of several genes in a heat map to adapt to TNT.	81.9% of the RDX (initial 40 mg L^−1^) was degraded in first 24 h, compared to non-adaptable organism to environmental stress the efficiency of removal is 3 times higher.	Several compromised genes in bacterial membrane lipid metabolism were upregulated (92 genes) and change composition and structure of membrane to tolerate such stress.	In 25 h of study only the initial and final profile after degradation were measured, the monitoring of strain develop in time remains unknown and predicted behavior is based on metabolomic results.
Microbial Community Dynamics during Acetate Biostimulation of RDX-Contaminated Groundwater [58].	Field evidence in bioaugmentation in metagenomics profiles and how bacterial abundance and geochemical properties change through 1 year of RDX and heavy metal pollution.	Constant degradation in time with maximum 2 µg L^−1^ achieve by biostimulation, in contrast with natural attenuation the dynamics of microbial will not be as efficient in remediation.	Microbial community profile change, dominant species dismiss in quantity and other species arise in response, the metagenomic profile evidence a shift in order to proliferate and mineralize RDX.	In one year, the dominance of *betaproteobacteria* is shift for *Bacteroides* and *deltaproteobacteria* arising from 10 to 60% and in specific genera in the last 6 months and arise of *geobacteraceae* showing a real time shift over microbial community.
Biostimulation and microbial community profiling reveal insights on RDX transformation in groundwater [59].	In field evidence of changes in military affected soil and ground water by biostimulator and microbial genomic profiles in high-explosives-machining facility.	4-NADB, TNT, RDX HMX and MNX were reduced to 400 ppm or less in al case achieving 70% of removal efficiency overall, in 35 days of study.	In response of biostimulation *proteobacteria* compared to other microbiota, gained domain in all samples showing better response of reducing toxicity in presence of other sources of nutrients to growth.	This study has no in field background of microbial change but instead exemplify the tendency of quick microbial community shift to adapt in field, with several monitored samples.
Enhanced phytoremediation of TNT and cobalt co-contaminated soil by *AfSSB* transformed plant [37]. *	Heat map of expressed genes before and after TNT and cobalt application, highlighting metabolism pathways.	TNT reduced to 1.23 mg kg^−1^ (up to 95% of efficiency uptake)	Upregulated genes in ROS scavenging to face against toxicity and stress of pollutant, specially focused on Cytochromes P450 and glutathione S-transferases, exist several damaged in DNA after TNT exposure.	15 days of monitoring several genes and proteomics with DNA studies, in overall in vitro study elucidate a probably case in field of adaptation to toxic pollutant.
Oxygen-insensitive nitroreductase bacteria-mediated degradation of TNT and proteomic analysis [60].	Proteome of nitro reductases and adaptation due to TNT exposure.	TNT degradation of 100% in 4 h	enriched in the pentose phosphate, glycolysis/gluconeogenesis, and amino acid metabolism pathway to stand against toxicity of TNT in vitro.	Insights in proteome change and the monitoring in short periods evidence proteomics as a tool for understanding byproducts toxicity, metabolisms and mechanism.

* An integrated research of degradation and metabolomics that focuses on relevant points for the use and explanation of phenomena related to degradation toxicity and integrative view of bioremediation application.

**Table 3:** Patents focused on explosive degradation and their technological innovation in chronological order.

Patent ID-Reference	Inventor	Year	Origin	Assignee	Brief Description	Innovation Related to Bio-Omics	Applications
DE10359610B4 [86]	Harald Claus, et al.	2003	Germany	Johannes Gutenberg Universitaet Mainz	Method for decontamination providing a nitro aromatic-degrading bacterial Isolates, *Klebsiella terrigena* HB (DSM 16101) or *Serratia* sp. M3 (DSM 16102), under aerobic or microaerophilic conditions resulting in 10% of the initial TNT concentration were reduced amino derivatives of TNT. Early patent expiration on 19 December 2023.	Isolated strains from contaminated soils and water, uptake and nitro reduction to cell nitrogen requirements	Germany ammunition manufacturing and Germany public use.
EP2242986B1 [87]	Thomas Smylie, et al.	2009	Germany -France	Orica International Pte Ltd.	Deactivating explosives using plant (parts, tissues or whole plant) and wherein physical growth of the plant. Contributes to physical breaking up of the explosive composition in a cartridge. Patent not in force and expiration in 2029.	An industrial and suitable phytoremediation approach	European union industry and military forces
US20110052537A1 [88]	A. Morrie Craig, collaborators	2011	United States of America	Oregon State University	A combined approach from plant uptake of TNT and RDX, a further plant digestion through ruminal anaerobic bacterial community achieving degradation of nitrogen compounds. The process of digesting the plant taking up the pollutant is done in the rumen; The ruminal anaerobic microbes degrade the remediable compounds and render them substantially nontoxic. This process is not toxic and harmless for the Clean-up of 90% and cost less than 112$ per cubic yard (anaerobic ex situ treatment), patent abandoned in 2011.	A complete ruminal anaerobic bacteria analysis through RT-PCR and culture media to assure degrading potential. Investigation of Diversity of Nitroreductase Gene. *	United states department of Agriculture and US military.
US8721813B1 [89]	Christian Clausen, et al.	2012	United States of America	University of Central Florida Research Foundation Inc UCFRF	Uses iron nickel (FeNi), iron palladium (FePd), and magnesium palladium (MgPd) provide in situ catalyst system for remediating and degrading nitro explosive compounds, a first nitroreduction step to further microbial, this is fee related and expired in 2027.	Mixed method of chemical low cost first denitration	US military.
CN103214058A [90]	Wei Zhixian, et al.	2012	China	North University of China	Uses a mixing powder or lanthanum nitrate, cerium nitrate, manganese chloride, ferric nitrate and acid, to assure degradation of TNT and RDX on wastewater also with UV radiation, patent is pending.	-	China industrial wastewater system.
EP2809402A1 [91]	Clint Brearly and collaborators	2013	Germany-France	Orica International Pte Ltd.	Microbial deactivation of explosive compositions, the use of inducer that stimulates an indigenous micro-organism to produce an enzyme that is able to degrade the explosive composition, up to 98% of effectiveness of removal currently not in force patent.	Using indigenous microorganisms and cooperation between European nations *	European union industry of ammunition or demolition and military forces
CN104195062A [92]	Dilibail t, et al.	2014	China	Xinjiang Normal University	Improvement of halophilic archaea and application thereof, *Haloarchaeon* sp. B13-RDX, with excellent resistance to recalcitrant compounds(explosives pesticides, etc.), expired-fee related and expire in 2034	Non common Archaea to assure degradation, metabolomics non dominant group	China Agriculture and industry
CN106497811B [93]	Ren Liwei, et al.	2015	China	Lianghua Biotechnology Beijing Co. Ltd.	Uses *Pseudomonas aeruginosa* CGMCC NO:10842 in soils degradation of TNT under hypersaline (ammonium or other salts) or stressful environmental conditions, this strain is tolerable and have high degradation capability, in general effectivity is up to 98.1%. The patent is active and expires in 2035	Isolated and preserved common strain in metagenomics of TNT degradation.	China biotechnology industry and military forces
US10351485B1 [94]	David L. Decker Joseph J. Grzymki	2017	United States of America Nevada	Desert Research Institute DRI Nevada System of Higher Education NSHE	Devices and methods for bio-passivating explosives (TNT, RDX, HMX), using a bioreactor with soils and water with microorganisms which are especially desirable candidates to safely consume and passivate explosive (bacteria fungi and others). This methodology involves schematic design of biopassivation reactors and water or additives supply for long terms activities to assure biological activity through time. Active patent with anticipated expiration in 2037.	Technical improvement in bioreactor fusing microbiological develop in other patents and the best process of passivation and degradation in multiuse chamber device. *	United states agricultural department military US forces and US industrial field.
CN109234246A [95]	Zhou Yang, et al.	2018	China	Institute of Chemical Material of CAEP	Use of flavo-enzyme that TNT open loop can be made to degrade, the mutant can be catalyzed the phenyl ring reduction of TNT and the first step of bioremediation. Patent in pending status.	Specific mutation on proteomics mapped enzymes for degradation (metabolomics)	China chemical industries
CN108277175A [96]	Ye Zhengfang, et al.	2018	China	Beijing Institute of Collaborative Innovation	2,4-dinitrotoluene (DNT) sulfonate efficient degrading bacterial strain *Microbacterium* sp. X3 CGMCC NO. 14586, involving a TNT derivative in production and biological degradation for complete removal of nitrogen energetic compounds. Removal efficiencies of 61.6% to 100% in dependence of the field application.	A technical biological innovation in gene and microbiological fields, focused on mid step derivative compounds of degradation.	Chemical industry and military forces
US20200222956A1 [97]	Scott Noland	2020	United States of America	Remediation Products Inc	A composition useful for removing energetic compounds (TNT, PENT, ADNT, and more) formulation include adsorbent and polymeric enhanced agent for breaking pollutants and polymers into smaller molecules for optimal degradation. Patent in pending status.	Non catalytic materials use for improve microbial and bioremediation towards formulation use of indigenous microbiome in situ	Chemical industry
CN112063545B [98]	Dang Kai, et al.	2020	China	Northwestern Polytechnical University	Aerobic *Pseudomonas* CGMCC No. 18373. Can grow by taking an energetic material such as CL-20 (explosive) as a unique nitrogen source. Efficiently degrades the energetic material in the environment leaving almost zero residual pollutant. Active patent.	Aerobic degradation by a only microbial agent, considering a metabolomics and genetic profiling	Chemical industry
CN116218712A [99]	Zhao Sanping, et al.	2022	China	Institute of Chemical Defense Chinese Academy of Military Sciences	Microbial consortia formulation for bioremediation of TNT using *Pseudomonas putida* T2, *Bacillus mycoides* T3 and *Bacillus amyloliquefaciens* T10. The process is improved through the synergistic effect of the three strains and specific inoculums reaching 96.4% of maximum removal. Pending patented process.	Application of biological science and modified GMO in Military fields. Ann biotechnology approach on military concern topics. *	China military Forces
CN116254195A [100]	Zhao Sanping, et al.	2022	China	Institute of Chemical Defense Chinese Academy of Military Sciences	Liquid microbial consortia *Pseudomonas aeruginosa* TC01, *Bacillus thuringiensis* TC05 and *Bacillus cereus* TC08 to be used in wastewater or industrial water. Rapid degradation of up to 97.3% of TNT present. Pending patent related to CN116218712A.	Application of biological science and modified GMO in Military fields, to assure safe environment. *	China military Forces, ammunition wastewater industry
CN116060434A [101]	Zhao Sanping, et al.	2023	China	Institute of Chemical Defense Chinese Academy of Military Sciences	Immobilized *Bacillus thuringiensis* in soils to assure degradation of explosives over time without perturbing microbiota. Low-cost immobilization matrix of corn stalk biochar and diatomite. Process is suitable for multiple uses, 99% of removal in 50 days. Pending patent.	Patenting all-important cost-effective process to remediate military ammunition environmental impact	China military Forces

* Patented applications and innovations correspond to progress on bioremediation processes, co-metabolic mechanisms, plant–microbe interactions and novel technologies developed for enhanced bioremediation through several studies, research and programs, and as the aim of a patent research and summary The environmental impact assessment of biologically enhanced treatments reached a new role in the sustainability and treatments of Hazardous Pollutants in Soil and Water.

## Data Availability

The data presented in this study are available upon request from the corresponding author.

## Supplementary Materials

The following supporting information can be downloaded at https://www.mdpi.com/article/10.3390/toxics12040249/s1 accessed on 1 November 2023. Figure S1: database google patents most relevant countries and assignees this will be relevant for exclusion parameters. Figure S2: database google patents most relevant countries and patent production over time this will be relevant for exclusion parameters.
