# Supplementary material for: Review of Explosive Contamination and Bioremediation: Insights from Microbial and Bio-Omic Approaches
Source: Toxics. 2024 Mar 29;12(4):249. doi: 10.3390/toxics12040249 (PMC11053648; doi:10.3390/toxics12040249)
Supplement: Supplementary file 1 [file toxics-12-00249-s001.zip › toxics-2757703-Supplementary.pdf]

Supplementary figures

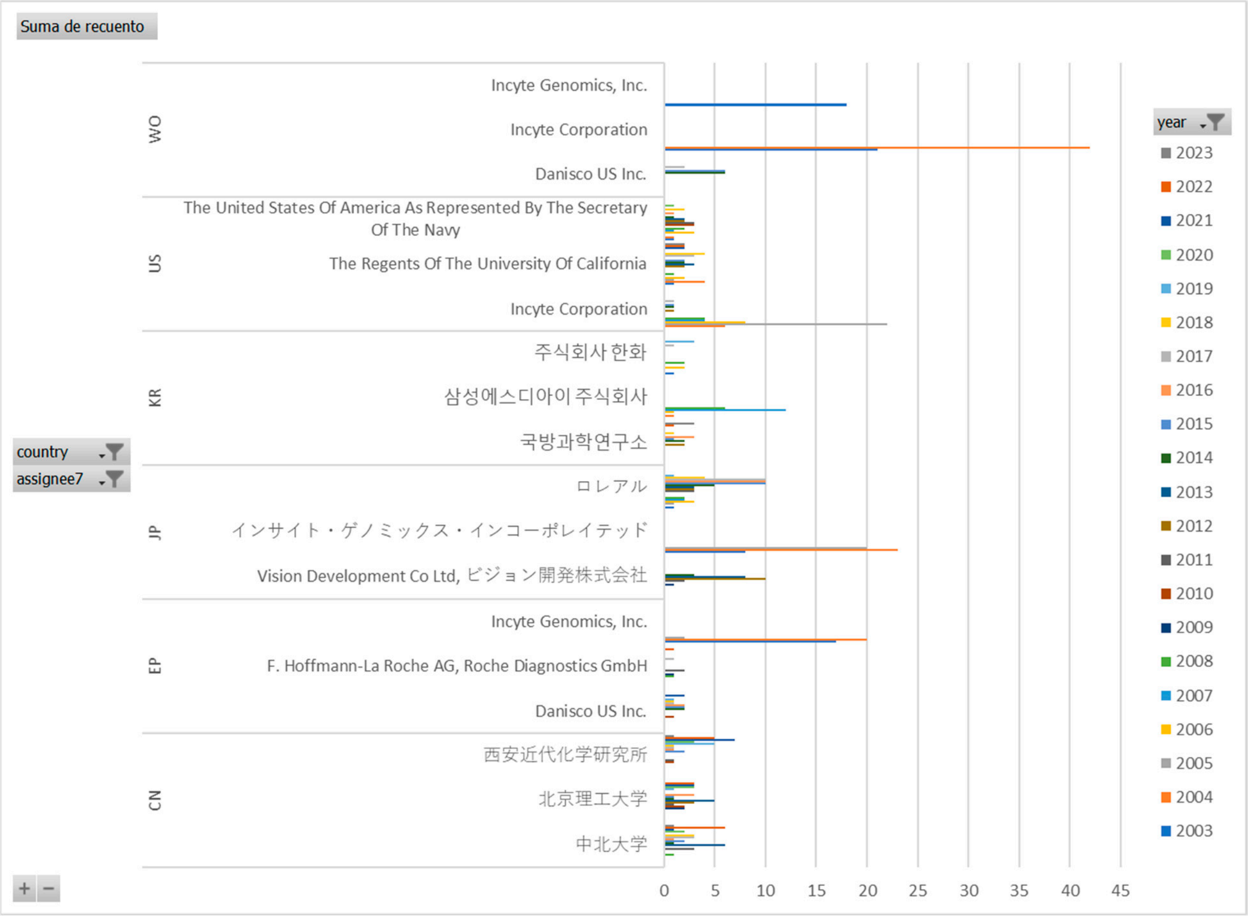

Supplementary Figure S1 first step of search approach includes the data base google patents most relevant countries and assignees this will be relevant for exclusion parameters, in the relevant information of assignees the military force related institutions and universities domain the best results of search, the database of patents is .

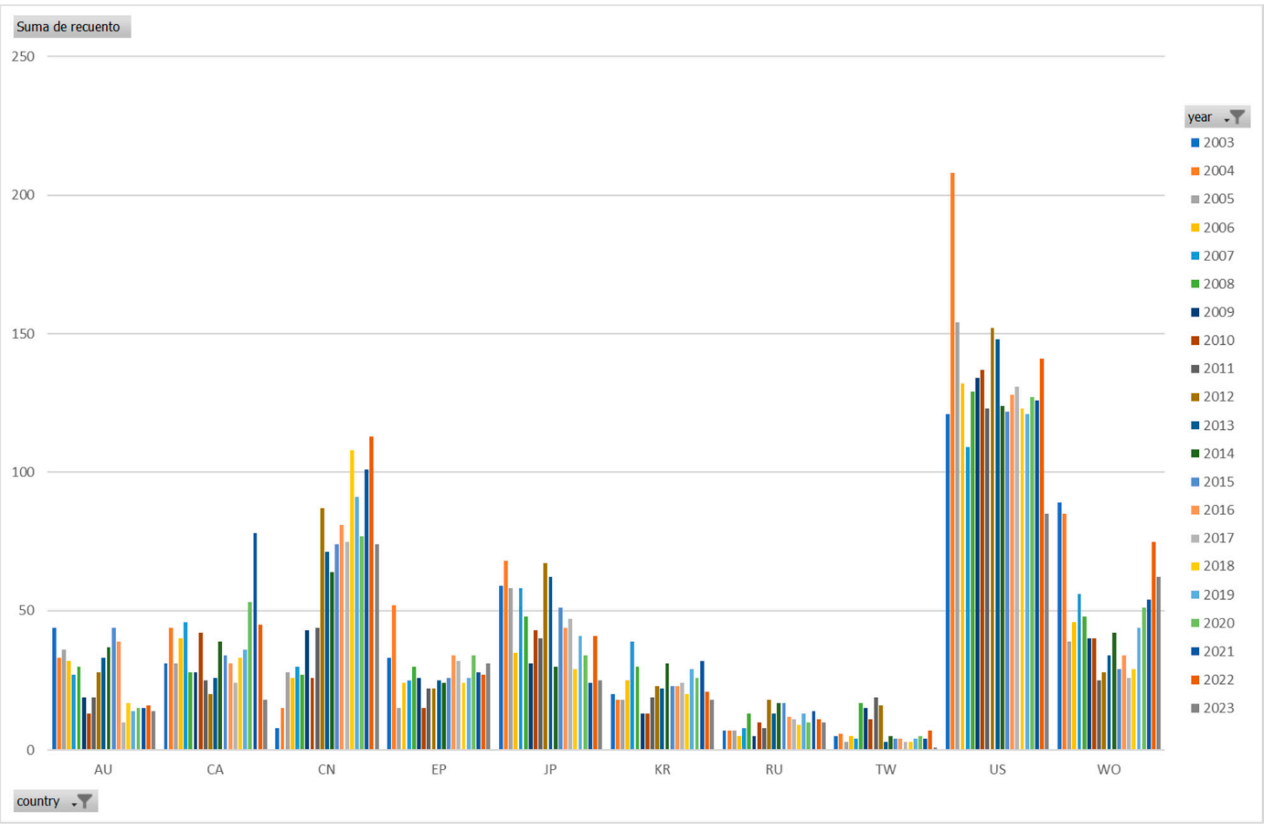

Supplementary Figure S2 first step of search includes data base google patents most relevant countries and patent production over time this will be relevant for exclusion parameters onto next steps, the patents will correspond to a general search and the best result where US, China ,World Intellectual property organization, Japan and European union, whit those criteria the relevant patents will be related to this countries
